# Clinical values of nuclear morphometric analysis in fibroepithelial lesions

**DOI:** 10.1186/s13058-024-01912-8

**Published:** 2024-11-11

**Authors:** Conrad Lee, Heilum Yip, Joshua J.X. Li, Joanna Ng, Julia Y. Tsang, Thomson Loong, Gary M. Tse

**Affiliations:** 1 Department of Anatomical and Cellular Pathology, State Key Laboratory of Translational Oncology, Prince of Wales Hospital, Prince of Wales Hospital, The Chinese University of Hong Kong, Ngan Shing Street, Shatin, NT Hong Kong SAR; 2 Department of Pathology, Tuen Mun Hospital, Tuen Mun, NT Hong Kong SAR

## Abstract

**Background:**

Fibroepithelial lesions (FELs) of the breast encompass a broad spectrum of lesions, ranging from commonly encountered fibroadenomas (FAs) to rare phyllodes tumors (PTs). Accurately diagnosing and grading these lesions is crucial for making management decisions, but it can be challenging due to their overlapping features and the subjective nature of histological assessment. Here, we evaluated the role of digital nuclear morphometric analysis in FEL diagnosis and prognosis.

**Methods:**

A digital nuclear morphometric analysis was conducted on 241 PTs and 59 FAs. Immunohistochemical staining for cytokeratin and Leukocyte common antigen (LCA) was used to exclude non-stromal components, and nuclear area, perimeters, calipers, circularity, and eccentricity in the stromal cells were quantified with QuPath software. The correlations of these features with FEL diagnosis and prognosis was assessed.

**Results:**

All nuclear features, including area, perimeter, circularity, maximum caliper, minimum caliper and eccentricity, showed significant differences between FAs and benign PTs (*p* ≤ 0.002). Only nuclear area, perimeter, minimum caliper and eccentricity correlated significantly with PT grading (*p* ≤ 0.022). For differentiation of FAs from benign PTs, the model integrating all differential nuclear features demonstrated a specificity of 90% and sensitivity of 70%. For PT grading, the nuclear morphometric score showed a specificity of 78% and sensitivity of 96% for distinguishing benign/borderline from malignant PTs. In addition, a relationship of nuclear circularity was found with PT recurrence. The Kaplan-meier analysis, using the best cutoff determined by ROC curve, showed shorter event free survival in benign PTs with high circularity (chi-square = 4.650, *p* = 0.031).

**Conclusions:**

Our data suggested the digital nuclear morphometric analysis could have potentials to objectively differentiate different FELs and predict PT outcome. These findings could provide the evidence-based data to support the development of deep-learning based algorithm on nuclear morphometrics in FEL diagnosis.

**Supplementary Information:**

The online version contains supplementary material available at 10.1186/s13058-024-01912-8.

## Introduction

Phyllodes tumors (PTs) and fibroadenomas (FAs) are both fibroepithelial lesions (FELs) of breast, derived from intralobular stromal tissues. While FAs are the most common benign breast lesions, PTs are rare, constituting 2.5% of all FELs. PTs can be further graded into benign, borderline and malignant based on stromal histological features. FAs mostly do not recur after complete surgical excision. In contrast, PTs may recur with an overall recurrence rate of 21%. While local recurrences may occur regardless of grade, distant recurrences (metastases) are seen almost exclusively in borderline/malignant PTs [1]. Grade progression can occur during local recurrence [2, 3]. Due to their variable clinical behaviors, a precise diagnosis and better prognostication are crucial to patient management.

The diagnosis and grading of PTs are based on histologic assessment of stromal features. However these features among different grades of PTs are overlapping and in a continuum, making a precise stratification of PTs into the three grades difficult. At the benign end of the spectrum, PTs may show similar features with FAs, especially cellular FAs. Biomarkers have been studied to improve FEL diagnosis [3] and differentiate between FAs and PTs. Significantly higher Ki67 expression was found in PTs compared to FAs [4]. Stromal CD10 expression was found to be higher in borderline/malignant PTs than benign PTs/FAs [5]. More recently, molecular analysis has been proposed for the identification and classification of FELs. Mutations, including *RARA*, *SETD2*, *FLNA*, *RB1*, *TP53* and *TERT* promoter, can be found in PTs but not FA [6–9]. In addition, mutations in cancer driver genes and multiple mutations occurred mainly in borderline/malignant PTs [6, 8].

With the rapid advance in whole slide imaging (WSI), digital pathology (DP) plays an increasingly important role in the pathology workflow. With the introduction of DP, measurement and summation of morphological properties have been made much easier. Moreover, it allows a higher degree of reproducibility and lowers inter-observer bias [10, 11]. The quantitative data from DP analysis allows the mass measurement and calculation to uncover new relationships and interactions. Assessment of nuclear morphology has long been a crucial aspect of histologic and cytologic diagnosis [12]. Alterations in nuclear morphometric features, with changes in size and shape, are commonly observed in cancers. Its objective evaluation has been suggested in prognostication and diagnosis in different cancer types [13–18].

Nuclear features are important for FELs diagnosis. Mitosis and nuclear pleomorphisms are integral components in the histologic examination for FEL diagnosis. In this study, we explore the clinical relevance of nuclear morphometric analysis in FELs classification. By applying nuclear morphometric analysis on standard routine immunohistochemical (IHC) slides, we examined various nuclear features in a series of FAs and PTs and evaluated their relevance in differentiation of FAs and benign PTs as well as PT grading. In addition, their association with PT recurrence was also investigated.

## Materials and methods

### Study cohort

Cases of mammary PT diagnosed with benign, borderline, and malignant grades were retrieved by computer search from the Prince of Wales Hospital (PWH) and North District Hospital (NDH) sample storage between the years 2001 to 2020, as well as Tuen Mun Hospital (TMH) from 1997 to 2012 under consent. There were 241 PT cases collected. Additionally, 59 FA cases were selected and retrieved from PWH at 2020. Two blocks of formalin fixed paraffin embedded (FFPE) samples containing representative areas were collected for each case if available.

Four-micron sections were prepared from each case. H&E stained slides were reviewed to confirm FEL diagnosis according to WHO criteria [1]. For PT, stromal histologic features, including stromal cellularity, nuclear pleomorphism, stromal overgrowth, mitotic rate and tumor border were reviewed. Tumor border was characterized as pushing, partial infiltrative or infiltrative. Mitotic activity was quantified by counting mitotic figures per 10 HPF (Nikon Labophot, field area 0.19mm^2^) over the stroma. Nuclear pleomorphism and stromal cellularity were graded as mild, moderate, or marked. Stromal overgrowth was defined as consisting only of stromal cells without the presence of epithelial element at a lower power field (Nikon Labophot, field area 1.9mm^2^) and was graded as absent, partial (present very focally) or present. Patients’ age, tumor size, and outcome data were retrieved from the medical records. Event-free survival was defined as no recurrence or metastasis occurred from the date of first PT diagnosis to the date of the last follow up or an event diagnosed. The study was approved by the Joint Chinese University of Hong Kong-New Territories East Cluster Clinical Research Ethics committee (CUHK-NTEC CREC).

### Immunohistochemical (IHC) staining

To exclude the non-stromal components, IHC staining of leukocyte common antigen (LCA) and cytokeratin (CK) was performed to highlight all leukocytes and epithelial components. A cocktail stain with a mixture of antibodies against LCA (clone 2B11 + PD7/26; DAKO, 1:300) and CK (clone AE1/AE3; DAKO, 1:200) was applied. The staining was performed using Optiview Universal DAB Detection Kit (Ventana, Arizona, USA) after deparaffinization, rehydration and antigen retrieval (Ventana CC1 ULTRA at 98 °C for 36 min) and counterstained manually by 50% Mayer’s hematoxylin for one minute.

### Slide scanning and image analysis

**Fig. 1:**
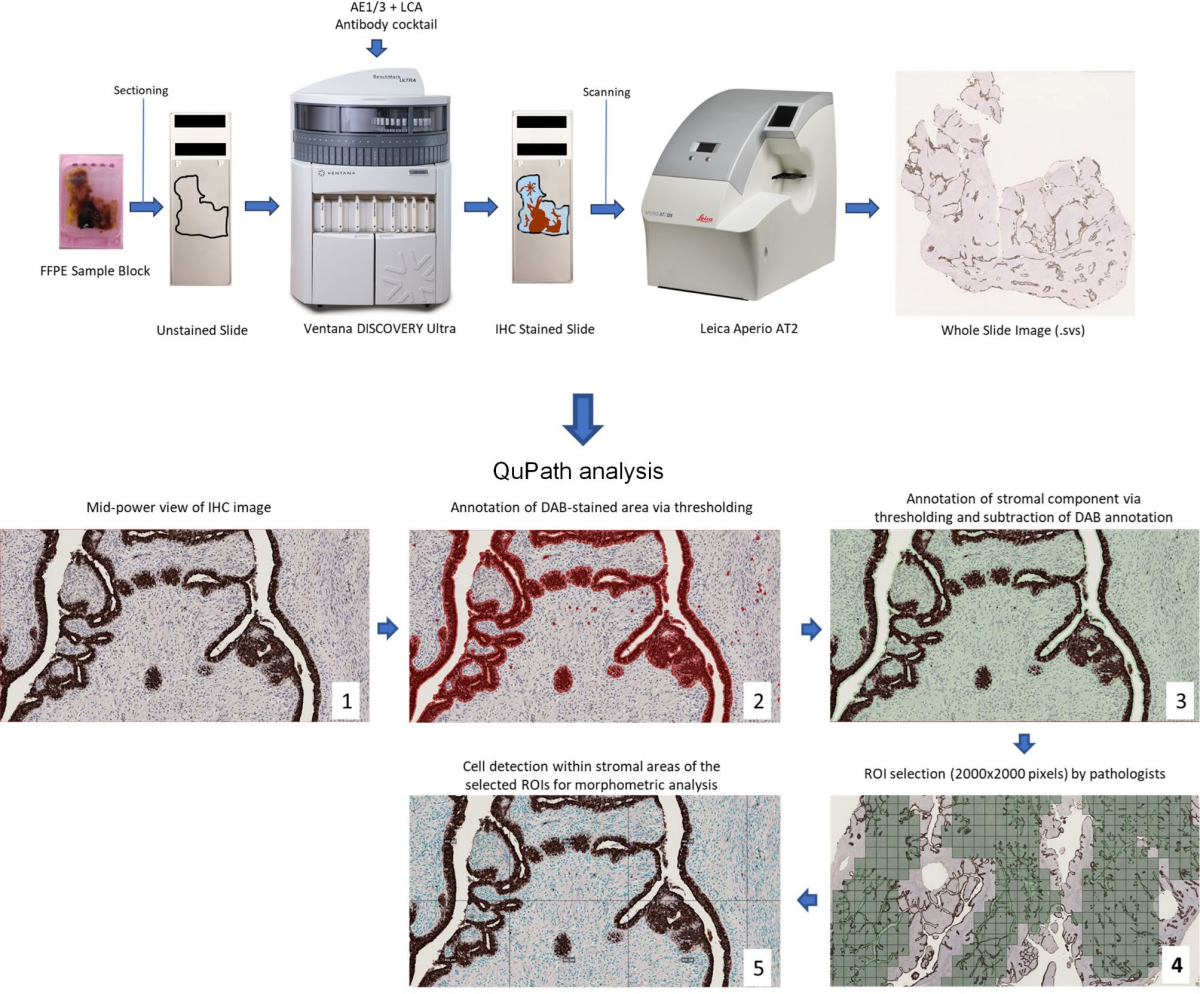
Schematic diagram showing workflow on IHC and digital analysis

Whole slide scanning was performed by the Leica Aperio AT2 (Leica Biosystems, Newcastle upon Tyne, UK) at 40X magnification (0.25 μm/pixel). Output images were in the format of SVS, a tiled tiff-based format created by Leica Biosystems. Image analysis was performed using QuPath (Version 0.4.3). The hematoxylin and DAB stains were separated by color deconvolution of the RGB images. Using the inbuilt QuPath threshold and watershed segmentation algorithm, nuclear segmentation was completed by automated extraction of nuclei throughout the whole scanned image based on hematoxylin optical density (OD). DAB threshold was set to filter out all the IHC stained epithelial components and leucocytes in the sections (Fig. 1). The parameters applied in the hematoxylin OD and DAB thresholds were shown in supplementary table S1. Only nuclei from DAB-negative cells in the stromal compartments were included in the subsequent nuclear morphometric analysis. The quality of nuclear segmentation and thresholding was counter-checked by a board-certified pathologist to ensure high accuracy in tumor nuclei selection and detection.

### Nuclear morphometric assessment

All scanned slides were divided into multiple tiles, of 2000 μm x 2000 μm in size. All the tumor areas in the stromal compartments were annotated on digitized slides by one pathologist and reviewed by another pathologist. All discrepancies were resolved by viewing and annotating the digitized slide together. All stromal areas were regarded as the region of interest (ROI). The nuclear morphological features, including nuclear area, perimeter, circularity, maximum and minimum caliper and eccentricity for each cell included in the ROI were evaluated by QuPath. The definitions of the nuclear morphometric parameters are as follows:

- Area: Area of a segmented nucleus in square pixels. It is expressed in a calibrated unit which is square micrometers (µm^2^);
- Perimeter: The length of the outside boundary of a segmented nucleus;
- Circularity: A function of both the nuclear perimeter and area it enclosed. It is calculated by the formula, 4π × area/(perimeter)^2^. A completely circular nucleus has a circularity of 1.00 and a value closer to 0.00 indicating a more elongated nucleus;
- Maximum caliper: The largest diameter measured in a particular nucleus;
- Minimum caliper: The smallest diameter measured in a particular nucleus;
- Eccentricity: The degree of deviation from a perfect spherical shape for a particular nucleus and it is expressed by a value between 0.00 and 1.00. The values of eccentricity are 0.00, 0.50 and 1.00 for a perfectly spherical shape, a regular elliptical shape and regular conical shape of a nucleus respectively.

The nuclear morphometric data were retrieved using python and R programming for further evaluation. The median, mean and the range of each parameter were recorded.

### Statistical analysis

Statistical analysis was carried out using SPSS software for Windows version 26.0 (SPSS Inc., Chicago, IL, USA). Mann Whitney U test was employed to compare the differences in nuclear parameters among different FELs, PT grades histopathological grading parameters, and recurrence. Receiver operating characteristic (ROC) analysis and areas under the curve (AUC) were adopted to determine the performance of different nuclear parameters in FEL diagnosis and determine appropriate cut-off values with the best sensitivity and specificity. Multivariate logistic regression analysis was carried out on the parameters with p value < 0.100 for constructing a model for FEL diagnosis based on nuclear parameters. Formulas for calculating the nuclear morphologic scores for (1) differential diagnosis of FA and benign PTs and (2) PT grade were constructed based on the estimated coefficient of the determined independent parameters: Nuclear morphologic score = β_0_ + β_1_ × _1_ + β_2_ × _2_+⋯βnXn.; where “n” is the number of constituting nuclear morphometric parameters, “X” is the value of a specific constituting nuclear morphometric parameter, “β_0_” is a constant of the logistic regression, and “β” is the driving coefficient for each morphometric parameter. Kaplan–Meier survival analysis was carried out for estimation of event-free survival of the two categories. The differences of the survival values were compared by using the Log-rank test. A p-value less than 0.05 was considered as statistically significant.

## Results

### Cohort features

Two hundred forty-one PT cases from 211 patients and 59 FAs were included. The patients’ age for PTs ranged from 16 to 86 (mean age = 42.8). The size of tumor ranged from 1.2 to 2.8 cm (mean size = 6.1). Paired primary and recurrent PTs were included for 12 patients. Four patients had two recurrences, including one patient with two PTs at different recurrent sites. Seven patients had one recurrence. Two PTs obtained at different sites from one patient were also included. One hundred forty-nine (61.8%) PTs were graded as benign, 66 (27.4%) as borderline and 26 (10.8%) as malignant.

### Comparison between different nuclear features among FEL and PT stromal histological features

Table 1 summarized the comparison between different nuclear morphologic features in different FELs and stromal features in PTs. The mean values with standard deviation of the six nuclear morphologic features (nuclear area, nuclear perimeter, nuclear circularity, maximum nuclear caliper, minimum nuclear caliper, and nuclear eccentricity) were presented. An increasing nuclear area was found with an increasing PT grade (*p* = 0.005). A higher nuclear perimeter and minimum nuclear caliper were found with a higher PT grade (*p* ≤ 0.022), but an opposite trend was found for eccentricity (*p* < 0.0001).

**Table 1:** Comparison between different nuclear features among FEL and PT stromal histological features

		Area	Perimeter	Circularity	Max Caliper	Min Caliper	Eccentricity
FEL Diagnosis	FA	14.9 ± 1.48	16.48 ± 0.73	0.67 ± 0.01	6.33 ± 0.27	3.30 ± 0.19	0.81 ± 0.01
	Benign PT	17.02 ± 2.06	17.35 ± 0.99	0.69 ± 0.02	6.65 ± 0.37	3.51 ± 0.25	0.80 ± 0.02
	Borderline PT	17.60 ± 2.20	17.53 ± 1.01	0.69 ± 0.02	6.65 ± 0.37	3.62 ± 0.26	0.78 ± 0.02
	Malignant PT	19.00 ± 3.25	18.18 ± 1.47	0.69 ± 0.02	6.84 ± 0.57	3.77 ± 0.34	0.78 ± 0.02
	p (PT grade)	**0.005**	**0.022**	*0.065*	0.469	**< 0.001**	**< 0.001**
	p (FA vs. Benign PT)	**< 0.001**	**< 0.001**	**< 0.001**	**< 0.001**	**< 0.001**	**0.002**
PT stromal features							
Border	Round	17.13 ± 2.17	17.38 ± 1.01	0.69 ± 0.02	6.64 ± 0.37	3.53 ± 0.27	0.79 ± 0.02
	Partial infiltrative	17.77 ± 2.59	17.67 ± 1.22	0.69 ± 0.01	6.72 ± 0.46	3.61 ± 0.28	0.79 ± 0.02
	Infiltrative	17.64 ± 2.42	17.56 ± 1.03	0.69 ± 0.02	6.66 ± 0.35	3.61 ± 0.30	0.79 ± 0.02
	p	0.360	0.600	0.879	0.989	0.257	*0.078*
Mitosis	Low	17.00 ± 2.01	17.34 ± 0.96	0.69 ± 0.02	6.65 ± 0.36	3.50 ± 0.24	0.80 ± 0.02
	Moderate	17.94 ± 2.42	17.69 ± 1.13	0.69 ± 0.02	6.70 ± 0.42	3.66 ± 0.27	0.78 ± 0.02
	High	18.59 ± 3.13	17.96 ± 1.43	0.69 ± 0.02	6.76 ± 0.53	3.74 ± 0.33	0.78 ± 0.02
	p	**0.003**	**0.026**	0.430	0.843	**< 0.001**	**< 0.001**
Pleomorphism	Low	17.17 ± 1.91	17.43 ± 0.92	0.69 ± 0.02	6.67 ± 0.35	3.53 ± 0.23	0.80 ± 0.02
	Moderate	17.15 ± 2.29	17.35 ± 1.06	0.69 ± 0.02	6.61 ± 0.39	3.55 ± 0.28	0.79 ± 0.02
	High	19.92 ± 3.04	18.54 ± 1.40	0.69 ± 0.02	6.94 ± 0.57	3.88 ± 0.29	0.78 ± 0.02
	p	**< 0.001**	**0.001**	**0.011**	**0.043**	**< 0.001**	**< 0.001**
Cellularity	Low	17.06 ± 1.94	17.37 ± 0.95	0.69 ± 0.02	6.66 ± 0.37	3.51 ± 0.23	0.80 ± 0.02
	Moderate	17.54 ± 2.56	17.53 ± 1.14	0.69 ± 0.02	6.67 ± 0.40	3.59 ± 0.30	0.79 ± 0.02
	High	17.89 ± 2.58	17.67 ± 1.19	0.69 ± 0.02	6.67 ± 0.45	3.66 ± 0.29	0.78 ± 0.02
	p	0.196	0.424	0.188	0.780	**0.017**	**< 0.001**
Overgrowth	No	17.39 ± 2.17	17.51 ± 1.03	0.69 ± 0.02	6.68 ± 0.38	3.57 ± 0.26	0.79 ± 0.02
	Partial	16.97 ± 2.24	17.26 ± 1.01	0.69 ± 0.02	6.60 ± 0.36	3.51 ± 0.29	0.79 ± 0.02
	Yes	18.00 ± 2.85	17.73 ± 1.29	0.69 ± 0.02	6.75 ± 0.50	3.63 ± 0.30	0.79 ± 0.02
	p	0.263	0.196	*0.054*	0.288	0.213	0.229

The data presented as mean ± SD

Bold value indicated statical significance

Comparing FAs and benign PTs, differences were observed in all nuclear morphologic features. The nuclear area, perimeter, minimum and maximum nuclear calipers were smaller in FAs than benign PTs, but its nuclear eccentricity was higher (*p* ≤ 0.002) (Table 1).

Regarding the PT stromal features, an increasing nuclear pleomorphism showed the same trend as PT grading in the associations with nuclear features (*p* ≤ 0.043). For stromal mitosis and cellularity, both were associated with an increased minimum nuclear caliper and a lower eccentricity (*p* ≤ 0.017). Additionally, a higher mitosis was also associated with increased nuclear area and perimeter (*p* ≤ 0.026). There were no differences in nuclear features across different tumour borders and stromal overgrowth (Table 1).

### Model building based on nuclear morphological features for FEL diagnosis

Next, we explored the possibility of applying nuclear morphologic features for FELs diagnosis. ROC analysis showed that all the assessed nuclear features, except maximum caliper, were useful to distinguish benign from borderline/malignant PTs, yielding AUC values ranged from 0.580 to 0.688 (*p* ≤ 0.038) (supplementary table S2). For distinguishing FAs from benign PTs, all nuclear features can be used for the differential diagnosis, showing AUC values ranged from 0.635 to 0.750 (*p* ≤ 0.002) (supplementary table S2).

**Fig. 2:**
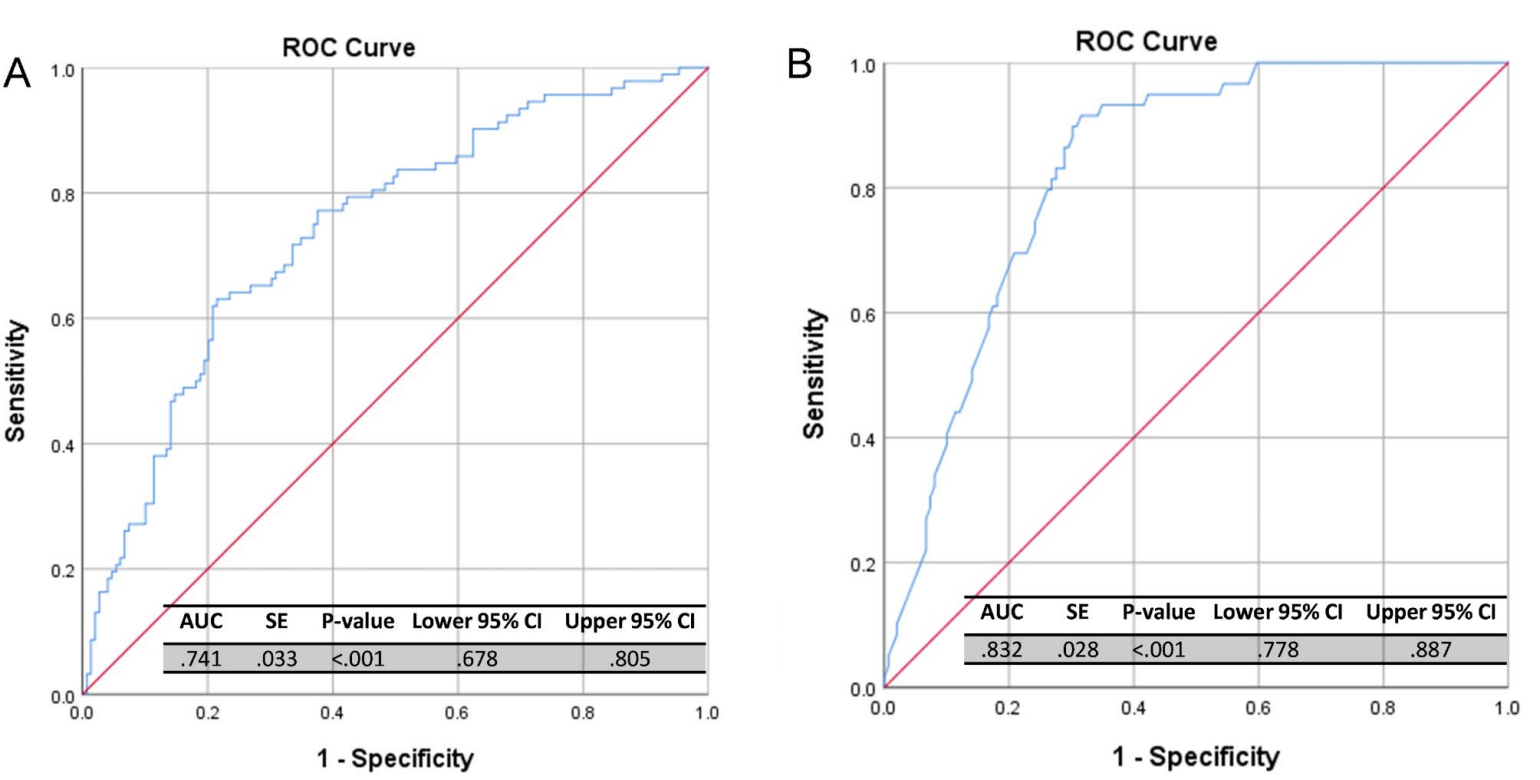
ROC curve of nuclear morphological score based on the logistic regression analysis in differentiating (**A**) benign PT from borderline/malignant PTs and (**B**) FA from benign PT

Multivariate logistic regression analysis was performed to identify independent nuclear features for FELs diagnosis. In PT grading, nuclear perimeter, minimum caliper, and eccentricity were found the independent significant features (*p* < 0.05). A PT grading diagnosis score was constructed based on the regression coefficients obtained for the significant nuclear morphologic features (Table 2). The PT grading nuclear morphology score outperformed individual parameters for distinguishing benign from PT of higher grades, with a higher AUC of 0.741 (Fig. 2A). The best cutoff of -0.2008 was determined from the ROC curve. A sensitivity of 77.4% and a specificity of 64.4% were found when applying the cutoff to discriminate benign from borderline/malignant PTs. A higher sensitivity (96.0%) and specificity (77.7%) can be achieved for identifying non-malignant PTs (Table 3).

**Table 2:** Model building based on multivariate logistic regression analysis of nuclear morphological features for FEL diagnosis

	Benign PT Vs Borderline/ Malignant PT		Benign PT Vs FA	
	Regression coefficient	p	Regression coefficient	p
Area	-	-	-3.881	< 0.001
Perimeter	2.381	< 0.001	-	-
Circularity	-20.392	0.075	-	-
Max Caliper	-	-	-	-
Min Caliper	-10.810	0.015	35.176	< 0.001
Eccentricity	-123.711	< 0.001	172.063	< 0.001
Constant	108.227	< 0.001	-197.039	< 0.001

**Table 3:** Diagnostic value of nuclear morphology score in FEL diagnosis

Nuclear morphology score	PT grading			FA diagnosis	
	Benign PT	Borderline PT	Malignant PT	FA	Benign PT
Low	117	28	6	6	104
High	32	38	20	53	45
	Benign PT	Non-Malignant PT		FA	
Specificity	64.4%	77.7%		90%	
Sensitivity	77.4%	96.0%		70%	

For the differentiation between FAs and benign PTs, nuclear area, minimum caliper, and eccentricity were found the independent significant features (*p* < 0.001 for all). Nuclear morphologic score for the differential diagnosis between FAs and PTs showed an AUC of 0.832 (Fig. 2B). Applying the best cutoff of -1.0596 determined from ROC curve, a sensitivity of 70.0% and a specificity of 90.0% were found for the diagnosis between FAs and benign PTs.

### Comparison of different nuclear features between PTs with and without recurrence

**Fig. 3:**
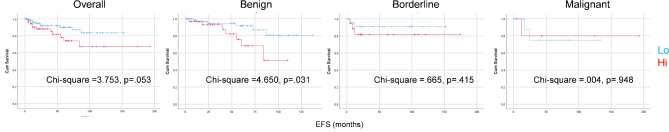
Kaplan meier analysis for event free survival of PT using circularity cutoff

Follow-up data were available for 169 PT patients. The mean follow-up period was 55 months (ranged from 1 to 194 months). There were 21 recurrent events of either local relapses (*N* = 17) or metastases (*N* = 4) recorded. Cases with recurrence showed a higher degree of circularity compared to those without recurrence (*p* = 0.017) (Table 4). No associations were found with other nuclear features. The AUC from ROC analysis of circularity for PT recurrence was 0.661, with a 95% confidence interval between 0.553 and 0.768 (Supplementary figure S1). The best cutoff of 0.698 was used to classify the PT cases into subgroups of low and high circularity. The Kaplan-meier analysis for event free survival showed that PTs with high circularity demonstrated a trend of poorer survival (chi-square = 3.753, *p* = 0.053). Interestingly, when the Kaplan Meier analysis was conducted on PTs with different grades, significantly shorter event free survival was found in benign PTs with high circularity (chi-square = 4.650, *p* = 0.031) (Fig. 3).

**Table 4:** Comparison between different nuclear features PT with and without recurrence

Event	Area	Perimeter	Circularity	Max Caliper	Min Caliper	Eccentricity
No	17.28 ± 2.28	17.41 ± 1.00	0.69 ± 0.02	6.63 ± 0.36	3.56 ± 0.28	0.79 ± 0.02
Yes	16.75 ± 1.98	17.09 ± 1.07	0.70 ± 0.02	6.50 ± 0.42	3.53 ± 0.23	0.78 ± 0.02
p	0.536	0.306	**0.017**	0.259	0.821	0.287

The data presented as mean ± SD

## Discussion

FELs of the breast encompass a wide range of lesions, from commonly encountered FAs to rare malignant PTs. Current classification relies on assessments of multiple histological features in a continuum. Grading based on these parameters has proven problematic due to their apparent unequal weighting [19]. Additionally, the overlapping features between different FELs render the precise cutoffs for diagnosis subjective, complicating accurate diagnosis and grading [20]. Inter-observer variability is pronounced in FEL classification, especially at the benign end of the spectrum [21]. This inconsistency undermines the diagnostic reliability, which is crucial for predicting prognosis and guiding appropriate treatment options. Nuclear atypia is a diagnostic hallmark in various cancers, playing a significant role in differentiating between benign and malignant tumors. With the advance in digital pathology, there is potential for a quantitative and objective assessment of nuclear morphological characteristics on hematoxylin and eosin (H&E) stained Sects. [22, 23]. Prior studies have demonstrated the utility of digital analysis features in distinguishing different mammary lesions [16, 24]. Here, we explored the application of digital nuclear morphometric analysis in FEL diagnosis. Our protocol, performing the analysis on an IHC stained section and using an open based software platform, makes it easily adaptable for routine clinical practice with minimal additional costs. This analysis generates sub-visual diagnostic information in an objective and reproducible manner, addressing the inconsistencies of manual interpretation and providing detailed data that enhances stratification in FEL diagnosis. Accurate pre-operative diagnosis of FA can prevent unnecessary surgeries. Nuclear features provide insights into both grading and prognosis. The risk of metastasis primarily limited to malignant PTs which require a more aggressive treatment. These results can also be instrumental in counseling patients about recurrence risks following surgical excision.

Our study revealed significant differences in all nuclear features, including area, perimeter, circularity, maximum caliper, minimum caliper and eccentricity, between FAs and benign PTs. Notably, these features, except maximum caliper and circularity, correlated significantly with PT grading. Models integrating the differential nuclear features showed better discriminatory powers than individual features as indicated by a higher AUC values. The nuclear morphometric score achieved a specificity of 90% and a sensitivity of 70% for differentiating FAs from benign PTs, while it demonstrated a specificity of 78% and a sensitivity of 96% for distinguishing benign/borderline PTs from malignant PTs. These findings underscore the potential of its application in classifying FELs. It can be more accurate than visual histopathological assessments, which are often subjective. In addition, a relationship between nuclear circularity and PT recurrence was found, suggesting that the application of digital morphometric analysis could extend to prognostic assessments for PTs. Stromal characteristics of atypia, mitoses, cellularity, overgrowth and nature of tumor borders are key parameters in FEL diagnosis. Although nuclear morphometric features correlated with PT grade, they were primarily associated with some diagnostic histological features only. Nuclear pleomorphism was defined by variation in size, shape and staining of the nucleus. The strong association of nuclear pleomorphism with nuclear morphometric, measuring the nuclear size (nuclear area and perimeters) and shape (minimum caliper, eccentricity and circularity), was to be expected. Mitosis and cellularity were also correlated with part of the nuclear features. Nuclear shape changes dynamically during cell division. Its size increases through cell cycle while its lamina which providing structural support undergo a transient disassembly during division [25, 26]. These events may lead to changes in nuclear morphometrics. Increased cellularity could lead to a different mechano-transduction, thus imposing changes onto the nucleus [27]. In PT grading, the analysis of stromal overgrowth and border could be less contentious. However, the identification of mitotic nuclei is a subjective and time-intensive task while the assessment of pleomorphism and cellularity may be hampered by inter-observer variability. Although it may have limitation in precisely classifying all borderline and malignant PTs, the nuclear morphometrics may be a valuable additional tool for PT grading. It is possible that its accuracy can be improved when combining with other histological features. Regarding the underlying molecular changes associated with nuclear morphometrics, there is no definitive association between nuclear shape and size and specific molecular alterations currently [28]. Notably, a unique association of *ARID1B* alterations with nuclear pleomorphism and not observed with other diagnostic histological features, was found in our previous analysis [29]. Given ARID1B’s role in chromatin remodeling and close relationship between chromatin remodelling and nuclear morphometrics [30], alteration in *ARID1B* could potentially be contributed to the changes in nuclear morphometrics in PT. For the differential diagnosis of FAs and benign PTs, useful features for PT diagnosis include the presence of stromal fronds, a higher degree of stromal cellularity, and periductal stromal accentuation [31]. However, these features may be underappreciated in the limited sample on core needle biopsy (CNB), complicating the distinction between FAs and PTs [20]. The nuclear morphometric analysis which focused on cellular features rather than tissue architecture, may be particularly useful for diagnosis on CNB.

Among the different histologic features for PT grading, stromal pleomorphism was shown to be an independent predictor of PT clinical behavior, having the highest hazard ratio [32]. Given the strong association of nuclear morphometry with stromal atypia, the relationship of nuclear feature and PT outcome was also investigated. Nuclear circularity was found to be a significant predictor for event-free survival, particularly in benign PT. A high score in nuclear circularity suggested a rounder tumor nucleus. Prior studies have demonstrated that adult bone marrow-derived mesenchymal stromal cells with less circular shapes exhibit restricted migration and poorer protrusion formation [33]. We speculated that PT stromal cells adopted a more circular nuclei might possess a greater migratory potential, leading to recurrences originating from such cells. PT stromal cells with round nuclei might resemble normal spindle cells at the surgical margin, posing challenges in assessing margin status and increasing the risk of false negatives. A positive surgical margin is a recognized risk factor for PT recurrence [2]. These hypotheses merit further validation. Establishment of benign and malignant PT cell lines have been reported recently [34]. It will be interested to investigate the relationship of nuclear circularity with migratory capacity of different PT cell lines using in vitro transwell migration assay. Moreover, the effect on migration after manipulating Lamin A/C expression which may alter nuclear circularity in PT cell lines can be tested. Additional validation studies with a prospective cohort can substantiate the correlation of nuclear morphological features in clinical management of PTs. With further validation, the nuclear morphometrics could be developed for risk stratification of PT patients.

In summary, by applying digital nuclear morphometric analysis, our study showed that nuclear morphometric parameters can differentiate between various FEL entities. Distinction between FAs and benign PTs were found in all nuclear features, with nuclear area, minimum caliper and eccentricity being independent predictive features. For PT grading, nuclear perimeter, min caliper and eccentricity were independent features for benign diagnosis. The models constructed based on the nuclear morphologic features yielded a specificity of 78% and sensitivity of 96% for the diagnosis of non-malignant PTs and a specificity of 90% and sensitivity of 70% for the diagnosis of FAs. A higher nuclear circularity value was associated with shorter event-free survival, particularly in benign PTs, indicating its potential prognostic significance. Although further studies with larger cohorts are necessary to validate the optimal cut-off values for clinical application of this nuclear morphometric analysis, the current findings could provide the evidence-based data to support the development of deep-learning based algorithm focused on nuclear morphometrics in FEL diagnosis.

## Electronic supplementary material

Below is the link to the electronic supplementary material.

Supplementary Material 1

## Data Availability

Data is provided within the manuscript or supplementary information files.
