# Supplementary material for: Clinical values of nuclear morphometric analysis in fibroepithelial lesions
Source: Breast Cancer Res. 2024 Nov 11;26:156. doi: 10.1186/s13058-024-01912-8 (PMC11552124; doi:10.1186/s13058-024-01912-8)
Supplement: Supplementary file 1 — Supplementary Material 1 [file 13058_2024_1912_MOESM1_ESM.docx]

**Table S1 Parameter setting for QuPath analysis and DAB thresholding**

| **Tissue area detection** |
| --- |
| Average channel : >210 [Resolution: low; smoothed: 1 µm] |
| **DAB detection** |
| DAB channel : >0.26 [Resolution: high; Smoothed: 3 µm] |
| **Cell detection** |
| Detection : Hematoxylin OD |
| Nuclear parameters |
| Background radius: 0 µm |
| Median filter radius: 2 |
| Sigma: 0.6 |
| Min Area: 5 |
| Max Area: 200 |
| Intensity parameters |
| Threshold: 0.24 |
| Max background intensity: 0.2 |
| Split by shape: True |
| Exclude DAB: True |
| Cell expansion: 0 |
| Include Nuclei: True |
| Smooth Boundaries: True |
| Make Measurements: True |

**Table S2 ROC analysis of nuclear morphological features for FEL diagnosis**

|  | Benign PT Vs Borderline/ Malignant PT | | | Benign PT Vs FA | | |
| --- | --- | --- | --- | --- | --- | --- |
|  | AUC | 95% CI | p | AUC | 95% CI | p |
| Area | .603 | .529-.678 | .007 | .750 | .680-.819 | <.001 |
| Perimeter | .580 | .505-.654 | .038 | .727 | .655-.800 | <.001 |
| Circularity | .546 | .472-.621 | .228 | .691 | .614-.767 | <.001 |
| Max Caliper | .508 | .433-.584 | .828 | .723 | .651-.796 | <.001 |
| Min Caliper | .649 | .578-.721 | <.001 | .713 | .640-.787 | <.001 |
| Eccentricity | .688 | .571-.806 | <.001 | .635 | .555-.715 | .002 |

**Figure S1 ROC curve of nuclear circularity in predicting PT recurrence**


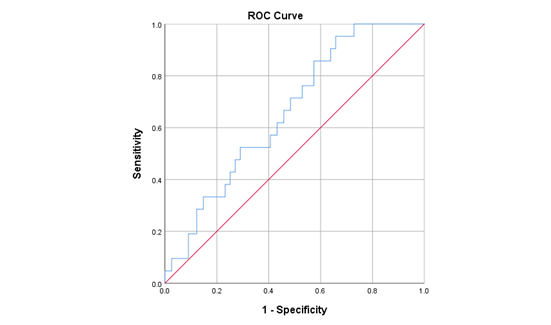


|  | AUC | SE | p-value | 95% CI Lower | 95% CI Upper |
| --- | --- | --- | --- | --- | --- |
| Circularity | .661 | .055 | .017 | .553 | .768 |
